# Childhood abuse and association with adult depressive symptoms among people with cardiovascular disease

**DOI:** 10.3389/fpubh.2023.1179384

**Published:** 2023-06-02

**Authors:** Ruoyun Yin, Yuan Yang, Lei Tang, Yujiao Chang, Fan Zhang

**Affiliations:** ^1^School of Public Health and Management, Chongqing Medical University, Chongqing, China; ^2^Department of Cardiovascular Medicine, The First Affiliated Hospital of Chongqing Medical University, Chongqing, China; ^3^Department of Infectious Disease, The First Affiliated Hospital of Chongqing Medical University, Chongqing, China

**Keywords:** childhood abuse, older adult depressive symptoms, cardiovascular disease, China, aging

## Abstract

**Background:**

To study the association between the total/different types of childhood abuse and adult depressive symptoms in people with cardiovascular disease (CVD).

**Methods:**

The subjects were people with CVD who continuously participated in the China Health and Retirement Longitudinal Study (CHARLS) life history survey and the 2018 wave of the CHARLS national baseline Survey. Multi-level logistic regression models were used to analyze the relationship between emotional neglect, physical neglect, physical abuse and adult depressive symptoms.

**Results:**

A total of 4,823 respondents were included in this study. The incidence of childhood abuse (existed emotional neglect, physical neglect or physical abuse) was 43.58% among people over 45 years old with CVD, which was higher than that of the general population (36.62%, *p* < 0.05). Adjusted model showed that overall childhood abuse was associated with adult depressive symptoms (OR = 1.230, 95%CI:1.094–1.383). Among different types of childhood abuse, only physical abuse was associated with depressive symptoms in adulthood (OR = 1.345, 95%CI:1.184–1.528).

**Conclusion:**

Compared with that of the general population, the incidence of childhood abuse in CVD population is higher. Physical abuse in childhood increased the risk of depressive symptoms in adulthood. It suggested that the occurrence of depressive symptoms was the result of related factors in the whole life course. In order to prevent the depressive symptoms, childhood abuse also needs to be considered. It is very important to identify and prevent the continuation of childhood abuse in time.

## 1. Introduction

Childhood abuse is a major public health problem worldwide. According to a survey conducted by the World Health Organization, about a quarter of the world’s population have been abused in childhood. In China, the situation of childhood abuse is also severe. Relevant studies have shown that the incidence of physical abuse of children in different areas is about 40.0% (1). Adverse Childhood Experience- International Questionnaire (ACE-IQ) classifies childhood abuse into five types: emotional neglect, physical neglect, emotional abuse, physical abuse and sexual abuse. Childhood physical abuse has always been the focus of early research. In recent years, the studies on emotional neglect and physical neglect is also increasing.

The health status of an individual is the result of the joint effect of all risk factors exposed over their life course (2). Life course epidemiology posits that an individual’s experiences in early childhood also influence their level of health in adulthood through specific pathways (3). In fact, multiple studies have found that experiences of abuse during childhood may have associations with physical and mental health in adulthood, especially mental health (4–7). Different types of childhood abuse tend to be present at the same time, and they can affect short-term and long-term mental health, alone or in interaction with each other. Studies have shown associations between childhood abuse and depressive symptoms (8), emotion regulation disorders (9), and interpersonal problems in adulthood (10). Childhood abuse may also simultaneously contribute to the development of adverse events such as alcohol dependence (11), obesity (12), drug use (13), and suicide (14).

Among the related researches, there are researches on childhood abuse and adult depressive symptoms. There is an association of childhood abuse with multiple types of depressive symptoms. Studies have investigated the association between childhood parental abuse and adult depressive symptoms. The results showed that parental abuse was associated with later chronic depressive symptoms (15). In addition, a Danish prospective cohort study found that childhood abuse increased the risk of moderate to severe unipolar depressive symptoms in adolescence and adulthood (16). A Dutch study of depressive symptoms in older adults revealed childhood abuse as a contributing factor to the course of depressive symptoms in later life (17). Overall, groups with a history of childhood abuse may have a higher risk of adult depressive symptoms than those who have not been abused in childhood. And the risk of this disease runs through the lifetime of the group.

People with cardiovascular disease (CVD) are at high risk of developing depressive symptoms. At the same time, people with CVD who are depressed may experience more severe depressive symptoms than depressed people without CVD (18). Therefore, it is crucial to study the factors that influence the occurrence of depressive symptoms in people with CVD. At present, the researches on depressive symptoms of patients with CVD often focused on the negative events or the influence of bad living habits at current stage (19, 20). For example, the risk of depressive symptoms might increase after the change of marriage condition. Related studies have found that individuals were more likely to have depressive symptoms after divorce and widowhood (21). Living habits such as drinking and smoking are not only risk factors for CVD, but also risk factors for depressive symptoms (20). After the occurrence of CVD, continuing to drink or smoke might further affect the cardiac function, and also aggravate the depressive symptoms. The influence of negative events related to childhood are less studied. Childhood abuse has been pointed out by some studies that it may be a risk factor for adult depressive symptoms in the general population (22–24). But is childhood abuse still an influencing factor of adult depression symptoms in CVD patients? Does every type of childhood abuse affect the occurrence of adult depressive symptoms in this population? After adjusting for influencing factors in adulthood and possible influencing factors in childhood [economic situation, parents’ depressive symptoms (8)], is this association still significant? These problems need to be further explored.

To date, fewer studies have examined the association of childhood abuse with adult depressive symptoms in population with CVD. And research is often limited to the effects of a single type of childhood abuse on adult depressive symptoms. We examined the association between childhood abuse and adult depressive symptoms in the Chinese middle-aged and older adults with CVD based on the China Health and Retirement Longitudinal Study (CHARLS) data. In our study, we studied the relationship between total childhood abuse and three specific types of childhood abuse (emotional neglect/physical neglect/physical abuse) and adult depressive symptoms.

## 2. Methods

### 2.1. Participants

Our research data were from the 2018 wave of the China Health and Retirement Longitudinal Study (CHARLS) conducted by Peking University. We also used CHARLS life history data. CHARLS is the first nationally representative longitudinal survey of people over the age of 45 in China. A stratified (by *per capita* GDP of urban districts and rural counties) multi-stage (county/district-village/community-household) population proportionate sampling random sampling strategy was adopted in CHARLS. Firstly, all county-level units in China were sorted (stratified) by region, within the region by urban district or rural county, and by GDP *per capita*. Then, 150 counties or urban districts were chosen with probability proportional to population size. For each county-level unit, 3 PSUs (villages and urban neighborhoods) are randomly chosen with probability proportional to population. Therefore, CHARLS is representative of people over 45 years old in China. In total, CHARLS conducted four national baseline surveys in 28 provinces, 150 districts and 450 villages/communities in 2011–2012, 2013, 2015, and 2018 respectively, while conducted a separate study on the life history of the surveyed population in 2014 (25). To date, the 2018 wave is the latest demographic baseline survey for CHARLS.

**Figure 1:**
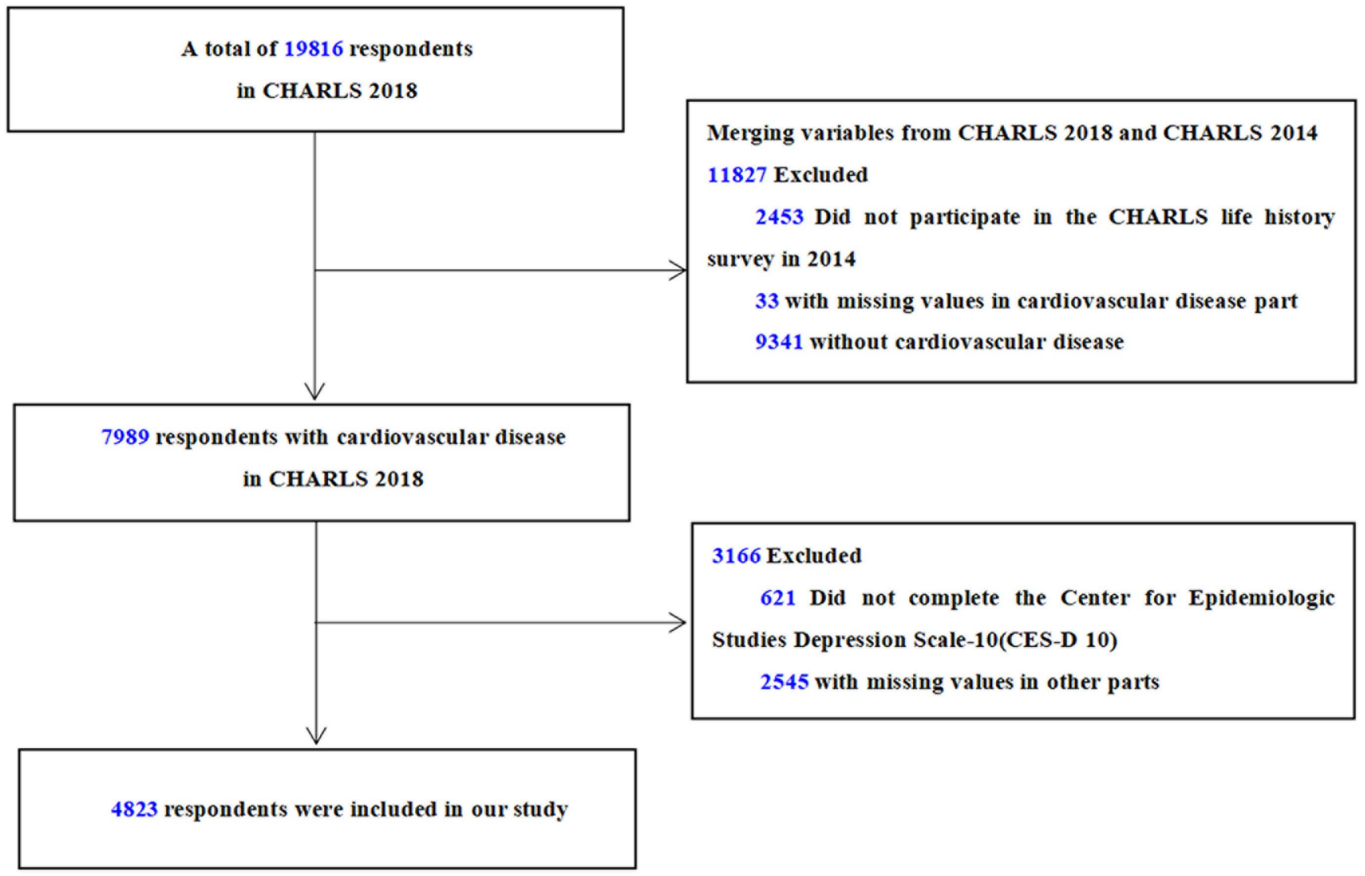
Flow chart of sample selection.

A total of 17,363 subjects participated in the CHARLS Life History Survey in 2014 and the CHARLS national baseline survey in 2018, of which 7,989 subjects had CVD. After excluding individuals who did not complete the Center for Epidemiologic Studies Depression Scale (CES-D, *n* = 621) at the time of the demographic survey or had missing values for other relevant study/control factors (*n* = 2,545), a total of 4,823 subjects were ultimately included in this study (Figure 1). In addition, we also considered the possible influence of missing values on the study population. The variables of the study population after deletion of missing values were compared with those of the whole population without deletion of missing values. There was no statistical difference among the variables (*p* > 0.05). Multiple interpolation (MI) was applied to the processing of missing values (Interpolation set = 10). The related statistical analysis results of the data after MI were consistent with those of the data after deletion of missing values. To sum up, it is feasible to delete the missing values directly. Our study strictly followed the Strengthening the Reporting of Observational Studies in Epidemiology (STROBE) guideline (26).

### 2.2. Measures

*Cardiovascular disease* CVD was determined by self-reports from participants. In the survey, the following two questions could reflect whether the interviewee suffer from CVD: ① Have you been diagnosed with hypertension by a doctor?; ② Have you been diagnosed with heart attack, coronary heart disease, angina, congestive heart failure, or other heart problems by a doctor? If participants’ answer to any of the above questions was “yes,” they would be judged to be suffering from CVD. Similar methods have been adopted in previous studies on CVD based on CHARLS (27, 28).

#### 2.2.1. Childhood abuse

ACE-IQ divides childhood abuse into five types: emotional neglect, physical neglect, emotional abuse, physical abuse and sexual abuse. Different types of childhood abuse were judged by specific questions in ACE-IQ. In recent years, more and more scholars in China have used ACE-IQ to study childhood abuse (29, 30). This study used ACE-IQ as a reference, and selected similar questionnaire items in the CHARLS life history survey to judge childhood abuse. Our research involved three types of childhood abuse: emotional neglect, physical neglect and physical abuse. Emotional neglect and physical neglect are judged by the following two questions: ① How much love and affection did your female guardian give you while you were growing up? ② How much effort did your female guardian put into watching over you? The answer was “rarely” or “never,” which meant there is abuse. Physical abuse was confirmed by combining the answers to the following three questions: ① When you were growing up, did your female guardian ever hit you?; ② When you were growing up, did your male guardian ever hit you?; ③ When you were growing up, how often did your brother or sister ever hit you? If the answer to one of the three questions was “often” or “sometimes,” the respondent would be judged to have physical abuse in childhood. For the overall childhood abuse, the interviewees were considered to have a history of childhood abuse as long as there was any type of childhood abuse.

#### 2.2.2. Adult depressive symptoms

CHARLS used 10-item Center for Epidemiologic Studies Depression Scale (CES-D 10) as a survey tool to study the depressive symptoms of the population (31). Previous studies have used a score of 10 as a cutoff value for significant depressive symptoms in CES-D 10 (32). The same judgment method was also adopted in our study. In the 2018 wave of CHARLS national baseline survey, people with CES-D scores of 10 or more were identified as having significant depressive symptoms.

#### 2.2.3. Control variables in the baseline survey

In order to ensure that the study was not affected by confounding factors, the subsequent analysis was adjusted according to demographic, behavioral as well as social factors. Data on common demographic variables such as gender, age (range: 45–97), and marital status were collected through participants’ self-reports. Among them, marital status was divided into three categories: married, unmarried and other marital status (separation, divorce and widowhood). Behavioral habits involved both smoking and drinking, which were set as binary variables. The decision was based on whether there was alcohol/smoking behavior more than once a month in the past 12 months.

The economic level of childhood and the mental status of parents in childhood are social factors related to depressive symptoms (33). Because the childhood of the surveyed population is too long ago, the reference value of their economic situation expressed by the amount of income is weak. CHARLS used a comparison with the surrounding population to determine the economic level of childhood of the participants. Our study divided participants’ childhood financial situation (before the age of 17) into three categories: “worse than others”, “same as others” and “better than others.” The mental state of parents in childhood was judged by the following two questions: ① During the years you were growing up, had your female guardian showed continued signs of sadness or depression that lasted 2 weeks or more? Was this problem of your female guardian, sadness or depression during all, most, some, or only a little of your childhood? ② During the years you were growing up, had your male guardian showed continued signs of sadness or depression that lasted 2 weeks or more? Was this problem of your male guardian, sadness or depression during all, most, some, or only a little of your childhood? If the answer to any question was “all” or “most,” the interviewees’ parents would be judged to be depressed.

### 2.3. Statistical analysis

Data analysis mainly included two parts: descriptive statistics and subsequent multi-level logistic regression analysis. The research factors involved are classified variables. In the part of descriptive statistics, we calculated the distribution of various research factors in the whole and different genders. Multilevel logistic regression models were used to analyze the relationship between different types and overall childhood abuse and adult depressive symptoms. For studies involving correction of multiple factors, multi-level logistic regression model is one of the commonly used statistical analysis methods (34). The analysis was adjusted based on three aspects: basic demographic characteristics, living habits, and depression-related factors. Multi-level logistic regression analysis included four models. Model 1 included only the overall/different types of childhood abuse into independent variables to observe the association between childhood abuse and adult depressive symptoms without adjustment. Model 2, model 3 and model 4 added demography related factors (sex, age, marital status), behavior habit factors (smoking status, drinking) and depression related factors (childhood economic level, childhood parents’ depressive symptoms) on the basis of model 1. Through the comparison between different models, the influence of different correction factors on the whole model was obtained. Odds ratio (OR) and 95% confidence interval (95%CI) were reported in the logistic model. Two-tailed *p* < 0.05 showed there was statistical significance. SPSS 20.0 was used for statistical analysis.

### 2.4. Ethics approvals

All the investigations involved in CHARLS were approved by the Institutional Review Board at Peking University. The Institutional Review Board (IRB) approval number of the main household survey is IRB00001052-11015. All the studies were conducted with the participants’ knowledge. CHARLS participants were asked to sign two informed consent forms before conducting the survey. This study is a secondary data analysis based on the public data of CHARLS. According to the relevant regulations, the secondary data analysis is not subject to ethical approval or informed consent.

## 3. Results

### 3.1. Participants’ characteristics in study

The subjects were Chinese people over 45 years old with CVD. In the 2018 wave of the CHARLS baseline survey, a total of 4,823 participants were included in our study. The mean (SD) age of the 4,823 respondents was 63.81 (9.58), of which 35.35% were male. In the survey of CES-D 10, the mean (SD) score of the participants was 9.57 (6.87), and 43.58% of them had a measurement score of at least 10 points. The CVD population in our study (43.58%) was more likely to have significant depressive symptoms than the total population in the 2018 CHARLS baseline survey (36.62%, *p* < 0.05). In the research on childhood abuse, 52.04% suffered abuse in childhood. Among the participants with a history of childhood abuse, the proportion of people who experienced physical abuse (30.96%) was higher than that of emotional neglect (20.82%, *p* < 0.05) and physical neglect (22.43%, *p* < 0.05). Of the participants who suffered childhood abuse, 66.45% experienced one type of abuse in childhood, 24.50% experienced two types of abuse, and 9.05% experienced three types of abuse in childhood. Table 1 showed the distribution of various research factors in the whole and different genders.

**Table 1:** Baseline characteristics of the participants (*n* = 4,823).

Variable	All (%)	Male (%)	Female (%)
Dependent variable			
CES-D-10 score			
<10	2,721 (56.42)	1,080 (63.34)	1,641 (52.63)
≥10	2,102 (43.58)	625 (36.66)	1,477 (47.37)
Childhood abuse			
Childhood abuse existence			
No	2,313 (47.96)	777 (45.57)	1,536 (49.26)
Yes	2,510 (52.04)	928 (54.43)	1,582 (50.74)
Emotional neglect			
No	3,819 (79.18)	1,383 (81.11)	2,436 (78.13)
Yes	1,004 (20.82)	322 (18.89)	682 (21.87)
Physical neglect			
No	3,741 (77.57)	1,350 (79.18)	2,391 (76.68)
Yes	1,082 (22.43)	355 (20.82)	727 (23.32)
Physical abuse			
No	3,330 (69.04)	1,080 (63.34)	2,250 (72.16)
Yes	1,493 (30.96)	625 (36.66)	868 (27.84)
Other factors			
Age			
<65	2,499 (51.81)	802 (47.04)	1,697 (54.43)
≥65	2,324 (48.19)	903 (52.96)	1,421 (45.57)
Sex			
Male	1705 (35.35)	1705 (100)	NA
Female	3,118 (64.65)	NA	3,118 (100)
Alcohol use			
No	3,953 (81.96)	1,012 (59.35)	2,941 (94.32)
Yes	870 (18.04)	693 (40.65)	177 (5.68)
Smoking status			
No	3,175 (65.83)	351 (20.59)	2,824 (90.57)
Yes	1,648 (44.17)	1,354 (79.41)	294 (9.43)
Marital status			
Married	4,013 (83.20)	1,495 (87.68)	2,518 (80.76)
Separated, divorced, widowed	786 (16.30)	187 (10.97)	599 (19.21)
Unmarried	24 (0.50)	23 (1.35)	1 (0.03)
Financial situation before 17			
Worse than others	1886 (39.10)	690.00 (40.47)	1,196 (38.36)
Same as others	2,435 (50.49)	861.00 (50.50)	1,574 (50.48)
Better than others	502 (10.41)	154 (9.03)	348 (11.16)
Childhood parents’ depression			
No	4,303 (89.22)	1,544 (90.56)	2,759 (88.49)
Yes	520 (10.78)	161 (9.44)	359 (11.51)

Before the multi-level logistic regression analysis, the collinearity test was carried out on the factors involved in the model in Tables 2, 3. The results showed that the research factors included in models in Tables 2, 3 were not collinear (VIF < 10). The follow-up analysis can be carried out normally.

**Table 2:** Association of childhood abuse existence and adult depressive symptoms in China: logistic model.

Variable	Model 1		Model 2		Model 3		Model 4		OR (95%CI)	*p*	OR (95%CI)	*p*	OR (95%CI)	*p*	OR (95%CI)	*p*
Childhood abuse existence								
No	1.0		1.0		1.0		1.0	
Yes	1.255 (1.119–1.407)	<0.001	1.272 (1.133–1.428)	<0.001	1.272 (1.133–1.427)	<0.001	1.230 (1.094–1.383)	0.001
Age								
<65			1.0		1.0		1.0	
≥65			0.853 (0.756–0.962)	0.009	0.844 (0.748–0.952)	0.006	0.842 (0.745–0.951)	0.006
Sex								
Male			1.0		1.0		1.0	
Female			1.527 (1.350–1.728)	<0.001	1.832 (1.528–2.196)	<0.001	1.834 (1.526–2.205)	<0.001
Marital status								
Married			1.0		1.0		1.0	
Separated, divorced, widowed			1.499 (1.275–1.761)	<0.001	1.480 (1.259–1.740)	<0.001	1.432 (1.215–1.687)	<0.001
Unmarried			2.878 (1.248–6.635)	0.013	2.808 (1.218–6.476)	0.015	2.401 (1.029–5.602)	0.043
Alcohol use								
No					1.0		1.0	
Yes					1.234 (1.042–1.469)	0.015	1.250 (1.053–1.484)	0.011
Smoking status								
No					1.0		1.0	
Yes					1.164 (0.977–1.386)	0.089	1.150 (0.964–1.373)	0.121
Financial situation before 17								
Worse than others							1.0	
Same as others							0.724 (0.639–0.821)	<0.001
Better than others							0.595 (0.484–0.733)	<0.001
Childhood parents’ depression								
No							1.0	
Yes							2.252 (1.857–2.732)	<0.001

**Table 3:** Association of childhood abuse and adult depressive symptoms in China: logistic model.

Variable	Model 1		Model 2		Model 3		Model 4		OR (95%CI)	*p*	OR (95%CI)	*p*	OR (95%CI)	*p*	OR (95%CI)	*p*
Emotional neglect								
No	1.0		1.0		1.0		1.0	
Yes	0.993 (0.854–1.154)	0.922	0.970 (0.833–1.129)	0.694	0.967 (0.830–1.127)	0.668	0.994 (0.852–1.160)	0.937
Physical neglect								
No	1.0		1.0		1.0		1.0	
Yes	1.143 (0.987–1.323)	0.074	1.134 (0.979–1.135)	0.094	1.136 (0.980–1.137)	0.091	1.113 (0.958–1.293)	0.161
Physical abuse								
No	1.0		1.0		1.0		1.0	
Yes	1.350 (1.194–1.528)	<0.001	1.419 (1.252–1.609)	<0.001	1.420 (1.252–1.610)	<0.001	1.345 (1.184–1.528)	<0.001
Age								
<65			1.0		1.0		1.0	
≥65			0.859 (0.762–0.969)	0.013	0.850 (0.754–0.979)	0.008	0.847 (0.750–0.958)	0.008
Sex								
Male			1.0		1.0		1.0	
Female			1.560 (1.378–1.767)	<0.001	1.873 (1.561–2.247)	<0.001	1.868 (1.553–2.247)	<0.001
Marital status								
Married			1.0		1.0		1.0	
Separated, divorced, widowed			1.512 (1.286–1.777)	<0.001	1.493 (1.269–1.755)	<0.001	1.443 (1.224–1.701)	<0.001
Unmarried			2.828 (1.225–6.531)	0.015	2.759 (1.195–6.368)	0.017	2.379 (1.017–5.564)	0.046
Alcohol use								
No					1.0		1.0	
Yes					1.239 (1.045–1.468)	0.013	1.252 (1.054–1.487)	0.010
Smoking status								
No					1.0		1.0	
Yes					1.163 (0.976–1.385)	0.092	1.151 (0.964–1.374)	0.121
Financial situation before 17								
Worse than others							1.0	
Same as others							0.731 (0.645–0.828)	<0.001
Better than others							0.599 (0.487–0.737)	<0.001
Childhood parents’ depression								
No							1.0	
Yes							2.220 (1.830–0.694)	<0.001

### 3.2. Association between the overall childhood abuse and adult depressive symptoms

In the unadjusted model 1, the presence of childhood abuse is associated with depressive symptoms in adulthood. Participants with a history of childhood abuse had a higher risk of depressive symptoms than those who did not experience abuse in childhood (OR = 1.255, 95%CI: 1.119–1.407). In other models that gradually added different corrective factors, total childhood abuse was always associated with adult depressive symptoms. Comparing model 2 with model 3, the OR of childhood abuse did not change after adding the related factors of behavior habits (smoking status, drinking). It showed that behavior-related factors had little impact on the model. After adding all the correction factors to model 4, childhood abuse still showed an association with adult depressive symptoms (OR = 1.230, 95%CI: 1.094–1.383), and its influence on the whole model was similar to that of drinking (OR = 1.250, 95%CI: 1.053–1.484). In addition, model 4 also showed that sex, age, marital status, alcohol use, economic status before the age of 17 and parental depressive symptoms were all influencing factors of adult depressive symptoms (*p* < 0.05). Table 2 showed the results of the multi-level logistic regression models that studied the association between the existence of overall childhood abuse and adult depressive symptoms.

### 3.3. Association between different types of childhood abuse and adult depressive symptoms

The multi-level logistical regression models for analyzing different types of childhood abuse and adult depressive symptoms consisted of four independent models. Model 1 analyzed the relationship between emotional neglect, physical neglect and physical abuse and adult depressive symptoms without being influenced by other factors. The results showed that only physical abuse was associated with depressive symptoms in the three types of childhood abuse (OR = 1.350, 95%CI:1.194–1.528). The conclusions of other three models were consistent with model 1. Compared with model 2, the OR of different types of childhood abuse in model 3 changed little. This showed that behavior-related factors had little impact on the whole model. In model 4 which included all relevant factors, physical abuse remained a risk factor for depressive symptoms in adulthood (OR = 1.345, 95%CI:1.184–1.528). Emotional neglect and physical neglect still did not show a correlation with the study outcome (*p* > 0.05). It can also be seen from model 4 that drinking, women, special marital status (separated, divorced and widowed), poor economic status in childhood (worse than others) and depressed parents in childhood all increased the risk of depressive symptoms in adulthood (*p* < 0.05, OR > 1). People over the age of 65 had a lower risk of depressive symptoms than those in the 45–65 range (OR = 0.847, 95%CI:0.750–0.958). Table 3 showed the results of the multi-level logistic regression models that studied the relationship between different types of childhood abuse and adult depressive symptoms.

## 4. Discussion

### 4.1. The present situation of childhood abuse in the middle-aged and older adults

The data of childhood abuse in this study came from the CHARLS life history survey in 2014, which was collected in the form of recall reports. We studied the childhood abuse of people over 45 years old. Childhood is defined as before the age of 17, which is consistent with many studies (35, 36). In the study population, the incidence of childhood abuse was 52.04%, lower than a study on childhood abuse of the older adults in Brazil (67.80%) (37). The survey of the World Health Organization (WHO) showed that about quarter of the world population suffered abuse during childhood. During the period 1940–1990, most of the population had a low level of education, a generally backward economic situation and a complex social background. This may be the cause of widespread childhood abuse. To some extent, it explains why the incidence of childhood abuse in the middle-aged and older adults is higher than that in the young population. In our study, we found that physical abuse (30.96%) was the most common type of childhood abuse, with a significantly higher incidence than emotional neglect (20.82%, *p* < 0.05) and physical neglect (22.43%, *p* < 0.05). But according to the Child Protective Service (CPS) system, emotional neglect is the most prevalent of all types of childhood abuse (38). In some families, corporal punishment is considered a means of educating children. Many parents do not treat this as the abuse of children. This may be the reason for the prevalence of physical abuse. Several studies have shown that different types of abuse tend to exist at the same time (38, 39). 33.55% of the population who suffered childhood abuse experienced more than two types of abuse in this study, which is similar with other studies.

### 4.2. Depressive symptoms in study population with CVD

In the middle-aged and older adults with CVD, the proportion of people with obvious depressive symptoms (CES-D ≥ 10) was 43.58%, which was higher than that of the normal population in the 2018 wave of the CHARLS national baseline survey (36.62%, *p* < 0.05). This result shows that people with CVD is a high-risk group suffering from depressive symptoms. Previous studies have found that depressive symptoms is more common in people with CVD (40), which is consistent with the results of this study. There is a very complex relationship between depression and CVD. People with CVD have a higher risk of depressive symptoms than the general population, while depressive symptoms is also a very common risk factor for the morbidity and mortality of CVD (18, 19, 41). The two cause effect each other. In addition, there are more and more studies on the co-disease of CVD and depressive symptoms in the older adults in recent years (42, 43). This shows that CVD and depressive symptoms related issues of the middle-aged and older adults are constantly being paid attention. Therefore, it is of great theoretical value and practical significance to study depressive symptoms in people over 45 years old with CVD in China.

### 4.3. Relationship between childhood abuse and adult depressive symptoms

Our study found a relationship between total childhood abuse and adult depressive symptoms. Total childhood abuse is defined as not less than one type of abuse during childhood. However, among different types of childhood abuse, only physical abuse was associated with depressive symptoms in adulthood. It is inferred that the experience of childhood abuse does not necessarily affect the occurrence of depressive symptoms in adulthood. The association shown by the analysis may be caused by physical abuse included in total childhood abuse. Several studies have shown that physical abuse/emotional neglect in childhood are associated with adult depressive symptoms (22, 44). A meta-analysis of 184 original studies found that abused individuals were 2.66 (95%CI, 2.38–2.98) to 3.73 (95%CI, 2.88–4.83) times more likely to develop depressive symptoms in adulthood, with the most significant correlation between the severity of depressive symptoms and emotional abuse and neglect (23). The results are supported by some studies (45). The relationship between physical abuse and adult depressive symptoms in our study is consistent with the results of domestic and foreign studies. But we found that there was no relationship between emotional neglect and adult depressive symptoms (*p* > 0.05). This difference may be caused by our different definition of emotional neglect. It is also possible that our research population is the middle-aged and older adults, and most of the research objects are more young groups. There is controversy about the relationship between physical neglect and adult depressive symptoms. Some studies have shown a relationship between childhood physical neglect and adult depressive symptoms (24, 46). In contrast, a longitudinal prenatal cohort study spanning 20 years found that physical neglect may have more to do with drug abuse / dependence and visual hallucinations than depression (47). At present, there are few studies directly analyzing the relationship between childhood physical neglect and adult depressive symptoms, and more related studies are needed to further verify the association between the two.

### 4.4. Suggestions to future studies

In our study, we found that total childhood abuse (existed emotional neglect, physical neglect or physical abuse), childhood physical abuse and adult depressive symptoms in patients with CVD were associated. This results could also be extended to the general population. We need to make it clear again that any type of abuse is a great mental and physical destruction for children. The impact of abuse is not limited to childhood, but continues into adulthood. According to the existing researches, childhood abuse is not only related to depressive symptoms, but also related to CVD, respiratory diseases and other diseases in adulthood (11, 44, 47). Future researches can further explore the short-term and long-term effects of childhood abuse on health status in different populations. Because of the sensitivity of sexual abuse question in middle-aged and old adults in China, the related effects of sexual abuse were not analyzed in this study. However, the impact of sexual abuse on children cannot be ignored, and its impact may be even worse than other types of childhood abuse. More researches are needed in the future to explore this point in depth. In addition, it is not enough to just conduct the retrospective analysis of childhood abuse. It is more important to take effective measures to identify and prevent the occurrence of childhood abuse. At present, many countries have adopted “mandatory reporting” systems to reduce the persistence of childhood abuse (48). But in China, there are no particularly effective preventive measures for children abuse. The improvement of the relevant prevention system is also worthy of our consideration.

### 4.5. Limitation

In the household questionnaire of the CHARLS National baseline Survey in 2018, disease data were collected from participants’ self-reports. There might be a certain deviation between the patient’s self-reported illness and the actual situation. Some potential CVD patients may be missed, such as those who have not been to the hospital for a long time.

In this study, 10 points were used as the CES-D 10 cutoff value to determine whether participants had significant depressive symptoms. Many studies have used similar methods to determine depressive symptoms (32, 49). However, some studies have shown that CES-D 10 is more suitable for evaluating the severity of depressive symptoms than as a diagnostic screening tool. When using 8 or 10 points as the cut-off value of CES-D 10, this screening method leads to good sensitivity (0.91/0.89) and poor specificity (0.35/0.47) (50). This means that some non-depressed patients might be misjudged as suffering from depressive symptoms. It affects the accuracy of the study to some extent.

We identified the different types of childhood abuse with reference to ACE-IQ. Its items are as close as possible to CHARLS items, but there are still some differences. This study was a secondary data analysis based on the CHARLS. It means that we could only analyze based on existing survey data. Although the Childhood Trauma Questionnaire (CTQ) may be better at identifying childhood abuse than ACE-IQ, it is difficult to find an one-to-one correspondence with the scale in the CHARLS. In this case, ACE-IQ is more suitable for the identification of childhood abuse. The questions of childhood abuse in CHARLS were also limited to parents or caregivers, and only investigated whether childhood abuse has occurred in this group. It also means that we could only identify childhood abuse existence, but not the specific degree of abuse. At present, studies on childhood abuse based on CHARLS have adopted this identification method (49). In addition, CHARLS aimed at investigating the Chinese population over the age of 45. In China, sex-related issues could be sensitive or even acute. The middle-aged and older adults might feel more ashamed or even offended in the face of such questions. Therefore, there is no question about sexual abuse in CHARLS. In fact, the researches on sexual abuse in China are more among young people. There is few research on this aspect among middle-aged and older adults. This is not only the limitation of this study, but also the content that needs to be further explored in this field.

## 5. Conclusion

We studied the total/different types of childhood abuse and its relationship with adult depressive symptoms in people over 45 years old with CVD. The results showed that the incidence of childhood abuse in CVD population is higher, compared with that of the general population. Physical abuse in childhood increased the risk of adult depressive symptoms. To study the depressive symptoms in middle-aged and older adults, we should pay more attention to their history of childhood abuse. At the same time, in order to protect children, it is also worth thinking about how to take effective measures to reduce the incidence of childhood abuse.

## Data availability statement

The raw data supporting the conclusions of this article will be made available by the authors, without undue reservation.

## Ethics statement

The studies involving human participants were reviewed and approved by the Institutional Review Board (IRB) approval number of the main household survey is IRB00001052-11015. Written informed consent to participate in this study was provided by the participants’ legal guardian/next of kin.

## Author contributions

YY and FZ conceived the study. The manuscript of this article was drafted by RY. RY and YY designed the writing strategies. YY, LT, and YC were involved in the statistical strategy for data analysis. All authors read and approved the final version of this article.

## Funding

This study was sponsored by Natural Science Foundation of Chongqing, China (Grant number: CSTB2022NSCQ-MSX0080).

## Conflict of interest

The authors declare that the research was conducted in the absence of any commercial or financial relationships that could be construed as a potential conflict of interest.

## Publisher’s note

All claims expressed in this article are solely those of the authors and do not necessarily represent those of their affiliated organizations, or those of the publisher, the editors and the reviewers. Any product that may be evaluated in this article, or claim that may be made by its manufacturer, is not guaranteed or endorsed by the publisher.
